# An interdisciplinary scoping review of the emerging impact of periocular skincare products on the ocular surface microbiome and tear film stability

**DOI:** 10.3389/fcimb.2026.1898798

**Published:** 2026-09-18

**Authors:** Precious Ochuwa Imokhai, Rebecca Metellus, McKenna Karsten, Maha Alhoda, Shikha Gandhi, Chiamaka Ogbuefi, Samantha Carranza, Jenny Zhang, Danny Lee, Amanda Brooks

**Affiliations:** 1 Rocky Vista University, Montana College of Osteopathic Medicine, Billings, MT, United States; 2 Geisinger Commonwealth School of Medicine, Scranton, PA, United States; 3 University of Washington School of Medicine, WWAMI, Moscow, ID, United States; 4 Royal College of Surgeons in Ireland, Busaiteen, Bahrain; 5 Arizona College of Osteopathic Medicine, Glendale, AZ, United States; 6 Rush College of Medicine, Chicago, IL, United States; 7 Wayne State University School of Medicine, Detroit, MI, United States; 8 Department of Medicine, University of California San Francisco, St. Mary’s Hospital, San Francisco, CA, United States; 9 Department of Research in Scholarly Activity, Rocky Vista University, College of Osteopathic Medicine, Ivins, UT, United States

**Keywords:** cosmetics, dysbiosis, immune modulation, meibomian gland dysfunction, ocular microbiome, periocular skincare, scoping review, tear film stability

## Abstract

**Background:**

The healthy ocular surface microbiome consists of *Actinobacteria*, *Proteobacteria*, and *Firmicutes* phyla, with *Corynebacterium*, *Propionibacterium*, and *Staphylococcus* as dominant genera. This microbial environment is essential for maintaining immune homeostasis and barrier integrity.

**Objective:**

To synthesize existing evidence on how periocular skincare and cosmetic products affect the ocular surface microbiome, tear film stability, and downstream ocular surface disease.

**Methods:**

A systematic literature search was conducted across PubMed, Cochrane Library, Embase, Web of Science, and Scopus using Boolean MeSH term strategies. Studies from 2010 to present evaluating microbial composition of the ocular surface and the effects of exogenous exposures (cosmetics, contact lenses, medications) were included.

**Results:**

Preservatives (benzalkonium chloride, parabens), surfactants, retinoids, and essential oils (tea tree oil/terpinene-4-ol) disrupt the ocular surface through non-selective microbial depletion, epithelial cytotoxicity, and lipid emulsification. These mechanisms contribute to meibomian gland dysfunction (MGD), blepharitis, and evaporative dry eye — all associated with characteristic dysbiotic microbiome shifts. Advanced sequencing technologies, including 16S rRNA gene sequencing and shotgun metagenomics, reveal taxon-level changes and functional alterations linked to inflammation and tear film instability.

**Conclusions:**

Periocular product ingredients represent an underappreciated source of ocular surface dysbiosis. Regulatory gaps under current FDA and MoCRA frameworks limit ingredient-level safety evaluation. Future longitudinal studies using functional metagenomics, metabolomics, and standardized microbiome-specific testing pipelines are urgently needed.

## 1 Introduction

The ocular surface encompasses the cornea, conjunctiva, eyelids, and the lacrimal and meibomian glands (Zilliox and Bouchard, 2023). Historically regarded as sterile, early culture-based studies yielded little to no microbial growth, most commonly detecting *Cutibacterium acnes (formerly Propionibacterium acnes)* or *Staphylococcus epidermidis* (McNatt et al., 1978). Advances in molecular techniques — particularly 16S rRNA gene sequencing — have since revealed that thousands of bacterial taxa colonize the ocular surface, with *Proteobacteria*, *Actinobacteria*, and *Firmicutes* as the dominant phyla (Katzka et al., 2021). Some debate remains as to whether detected organisms represent contamination from adjacent sites such as the nasal mucosa.

The ocular surface is a low-biomass environment, containing approximately 0.06 bacteria per conjunctival cell — compared to 10 bacteria per gut epithelial cell (Zilliox and Bouchard, 2023). Despite continuous environmental exposure, antimicrobial defenses provided by the tear film limit microbial proliferation. In mouse models, resident commensals such as *Corynebacterium mastitidis* inhibit pathogenic organisms by recruiting neutrophils (Chowdhary et al., 2023). Alterations in microbial diversity and composition have been linked to diseases including Stevens–Johnson syndrome and fungal keratitis (Katzka et al., 2021), suggesting dysbiosis may contribute to ocular surface disease.

There is growing interest in how periocular exposures — including cosmetic agents, skincare products, and ophthalmic medications — may alter this delicate ecosystem. Sodium hyaluronate eye drops have been shown to change the ocular surface microbiome, and mascara wands can harbor microbial contamination (Zhong et al., 2022; Sullivan et al., 2023). The objective of this scoping review is therefore to synthesize knowledge about the healthy ocular surface microbiome, how periocular agents affect its composition or function, what diseases are linked to ocular dysbiosis, and the experimental models and sequencing methods used to study these relationships.

## 2 Methods

A systematic literature search was conducted using PubMed, Cochrane Library, Embase, Web of Science, and Scopus. MeSH (Medical Subject Headings) terms were deployed with Boolean operators: [“ocular surface microbiome” OR “eye microbiota”] AND [“cosmetics” OR “skincare” OR “topical agents” OR “periocular”]. Inclusion criteria encompassed studies evaluating microbial composition of the ocular surface, including observational studies (case reports, cohorts, cross-sectional), experimental studies (randomized controlled trials), and systematic reviews published from 2010 to present. The search also targeted exogenous exposures (cosmetics, lenses, medications) and studies using 16S rRNA, shotgun metagenomics, culture-based, or proteomics methods. Non-English, non-peer-reviewed articles, and studies focused exclusively on the gut or skin microbiome were excluded.

The screening process involved review of abstracts for relevance and criterion fit, followed by full-text analysis. Screening was conducted systematically, focusing on study objectives, type, population, publication date, disease progression, and conclusions. Sections were allocated collaboratively among authors covering: characteristics of the ocular microbiome, cosmetic product effects, experimental models, and implications for future research. Limitations include potential selection bias and variability in study design, sample size, and sequencing methodology. A PRISMA (Preferred Reporting Items for Systematic Reviews and Meta-Analyses) flow diagram (**Figure 1**) details the search and selection process.

**Figure 1:**
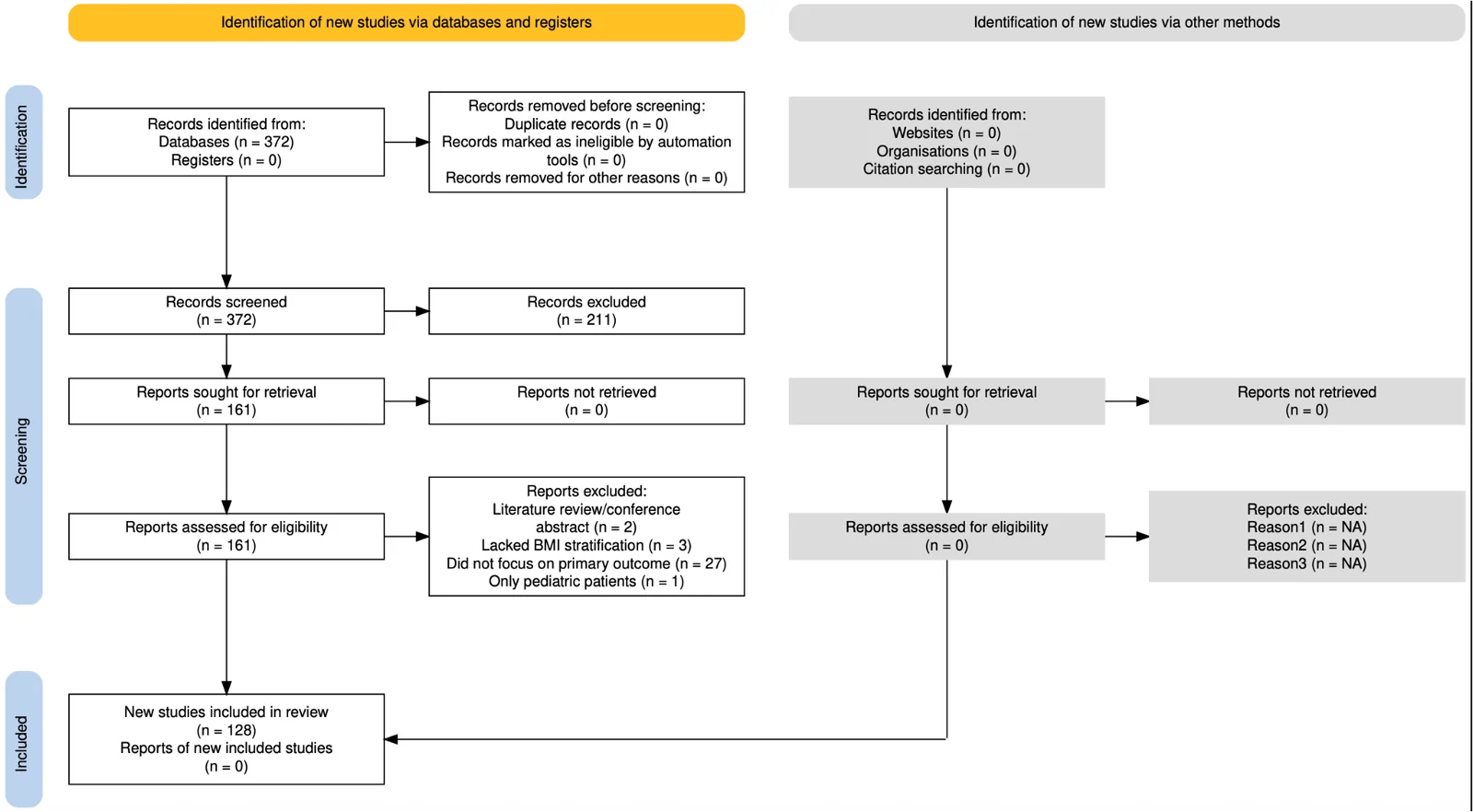
PRISMA flow diagram for search criteria and study selection.

### 2.1 Evidence categorization

Because the included literature spanned heterogeneous study designs, a formal meta-analysis or quantitative risk-of-bias score was not appropriate, consistent with established scoping-review methodology (Tricco et al., 2018). Instead, findings were qualitatively categorized into three evidence tiers to convey the strength and translational maturity of each claim:

1. Clinical evidence, human observational or interventional studies reporting microbiome or ocular-surface outcomes
2. Preclinical/*in vitro* evidence, cell-culture, *ex vivo*, or animal studies establishing biological plausibility and mechanism
3. Hypothesis-generating observations, single case reports, indirect associations, or mechanistic inferences not yet tested at the ocular surface. This tiering is reflected in the final column of **Table 1** and is used throughout the Results to distinguish established clinical associations from mechanistic or preliminary findings.

**Table 1:** Ingredient-level disruptors of the ocular surface and ocular microbiome.

Ingredient class	Specific agent	Proposed dysbiosis mechanism	Key findings	Level/Type of evidence	Reference
Preservatives	Benzalkonium chloride (BAK)	Non-selective microbial depletion; disruption of tear film stability	Increased gram-negative bacteria; direct cytotoxicity of conjunctival epithelial/goblet cells	Clinical (observational) and *in vitro*	Chang et al., 2022; Hedengran et al., 2021
	Methylparaben, Ethylparaben, Phenoxyethanol	DED-associated dysbiosis	Meibomian gland dysfunction	*In vitro* (preclinical)	Wang et al., 2020
Surfactants	Disodium cocoamphodiacetate (DC)	Corneal epithelial damage; compromise of protective barrier	Decreased protective proteome; increased inflammatory bacteria	*In vitro* and clinical observational	Wu et al., 2021; Schlegel et al., 2023
	Sodium Lauryl Sulfate (SLS)	Emulsification of ocular surface lipids; tear film disruption	DED-associated dysbiosis	*In vitro* (mechanistic)	Magny et al., 2020
Retinoids	Retinol, Tretinoin, Adapalene, Isotretinoin	Meibomian gland dysfunction	DED-associated dysbiosis	Clinical (observational)	Bayhan et al., 2016; Zakrzewska et al., 2023
Essential Oils	Tea Tree Oil (Terpinene-4-ol)	Non-selective antimicrobial effects; DED-associated dysbiosis	Decreased meibomian gland epithelial cells	*In vitro* and case report	Chen et al., 2020; Tharmarajah and Coroneo, 2021; Tighe et al., 2013; Wang et al., 2024
Direct Contamination	Expired mascara and eye cosmetics; product migration	Direct introduction of pathogenic organisms; product migration into tear film	Contamination with S. aureus and P. aeruginosa	Observational	Giacomel et al., 2013; Goto et al., 2010

This table summarizes ingredient-level disruptors, specific agents, and their proposed impact on the ocular surface and microbiome. The fifth column “Level/Type of Evidence”, indicates the predominant tier of supporting evidence for each entry (clinical, preclinical/*in vitro*, case report, or observational) to distinguish established clinical risks from purely laboratory findings. Key mechanistic effects suggest cosmetic and skincare ingredients disrupt the ocular surface epithelium, tear film stability, and contribute to dry eye disease (DED); the visual pathways for these interactions and their downstream clinical timelines are illustrated schematically in **Figures 7** and **Figure 8**, respectively.

## 3 The healthy ocular surface microbiome

The updated bacterial nomenclature proposed by Scholz and Kilian is used throughout this review. In 2016, the cutaneous members of the genus *Propionibacterium* were reclassified into the new genus Cutibacterium based on the whole-genome phylogenetic analyses. *Propionibacterium acnes* is now designated *Cutibacterium acnes*, while other cutaneous species such as *P. avidum* and *P. granulosum* were similarly reassigned. Although the genus name changed, these organisms remain within the *Propionibacteriaceae* family (Scholz and Kilian, 2016).

The ocular surface barrier relies on a specialized low-biomass microbial community responsible for regulating immune defense and stabilizing the tear film. *Corynebacterium mastitidis*, a conjunctival commensal, drives interleukin-17 (IL-17) production by γδ T cells, recruits neutrophils, and triggers secretion of antimicrobial peptides (S100A8/A9) into the tear film — protective against *Candida albicans* and *Pseudomonas aeruginosa* (St Leger et al., 2017). Disruption of this community leads to declining IL-17 levels and heightened infection risk (St Leger et al., 2017).

Metagenomic profiling has shown that abundance of *Firmicutes* correlates positively with tear proteins involved in lipid metabolism (FABP5, FABP7, and lipocalin-1), suggesting commensals contribute to tear lipid layer stability and may protect against dry eye (Zysset-Burri et al., 2021).

Although several studies have characterized the microbial composition of the healthy ocular surface, there remains no universally accepted healthy ocular microbiome baseline. Consequently, reported microbial profiles should be interpreted as population-level trends rather than a definitive healthy ocular microbiome baseline. The most repeatedly isolated genera from both culture and sequencing are *Staphylococcus* (46.5%), *Cutibacterium* (formerly *Propionibacterium)* (34.9%), *Micrococcus* (24.8%), and *Corynebacterium* (6.2%) (Ozkan et al., 2017). The ocular surface’s microbial biomass is up to 150-fold lower than facial skin, making it prone to contamination and amplification biases in sequencing (Salter et al., 2014).

Despite growing interest, critical knowledge gaps persist. No single OTU (operational taxonomic unit) has been detected across all participants at all time points, and genera such as *Corynebacterium* and *Staphylococcus* appear in only ~40% of participants (Ozkan et al., 2017). Most studies focus on taxonomic composition rather than microbial function. Longitudinal dynamics are poorly understood, and host factors such as sex, climate, and contact lens use remain inconsistently controlled across studies, limiting generalizability.

## 4 Cosmetics and skincare-related alterations in ocular microbiota

Using cosmetics in the periocular region is a near-universal practice. Giacomel et al. (2013) demonstrated that 70.5% of surveyed participants reported using expired cosmetics including mascara and eyeliner. Microbial analysis of mascara samples revealed high abundances of *Staphylococcus aureus* and *Pseudomonas aeruginosa* — organisms not native to the ocular surface and thus potentially dysbiotic (Giacomel et al., 2013). Goto et al. (2010) further showed that eyeliner and mascara can migrate directly into the tear film, exposing the ocular surface to both pathogens and chemical ingredients.

Such migration may shift the microbiome from protective commensals — coagulase-negative *Staphylococcus* and *Corynebacterium* spp. — toward potentially pathogenic organisms such as *S. aureus* (Watters et al., 2017). **Table 1** summarizes the key ingredient-level disruptors identified in the literature and includes an evidence-tier classification summarizing the strength of available evidence for each exposure.

### 4.1 Preservatives

Benzalkonium chloride (BAK), a quaternary ammonium compound used in eyeliners, mascaras, and eye creams, disrupts microbial lipid membranes non-selectively, depleting commensals alongside pathogens (Magny et al., 2020). Chang et al. (2022) documented increased gram-negative microbial predominance in patients using BAK-containing eye drops. *In vitro* evidence demonstrates cytotoxic effects on conjunctival epithelial and goblet cells in a time-dependent manner (Hedengran et al., 2021). Other preservatives — methylparaben, ethylparaben, and phenoxyethanol — have caused atrophy of human meibomian gland epithelial cells (HMGECs) under *in vitro* conditions (Wang et al., 2020), implicating them in MGD-associated dysbiosis.

Although preservative-free formulations might seem an obvious solution, their complete removal decreases product stability and increases pathogen risk. In ophthalmology, avoidance of preservative-containing eye drops in patients with dry eye or ocular surface inflammation is recommended (Gomes et al., 2017), but no equivalent guidance exists for cosmetic products, which lack equivalent regulatory oversight.

### 4.2 Surfactants

Surfactants in periocular cleansers, eye makeup removers, and micellar waters can damage the corneal epithelium at concentrations below those used in commercial formulations. Disodium cocoamphodiacetate (DC) significantly inhibited corneal epithelial proliferation and viability even at sub-cosmetic concentrations (Wu et al., 2021). Sodium lauryl sulfate (SLS) emulsifies phospholipids and sphingolipids essential for tear film stability and innate immune function (Magny et al., 2020). DED caused by surfactant-induced lipid disruption is itself associated with a characteristic dysbiotic profile featuring elevated *Corynebacterium tuberculostearicum* and *Propionibacteriaceae*, along with shifts in *Acinetobacter johnsonii* that reduce protective protein production (Schlegel et al., 2023).

### 4.3 Retinoids

Retinol, tretinoin, adapalene, and isotretinoin are widely used in anti-aging and dermatologic formulations marketed for the periocular region. Patients using prescribed retinoids for acne vulgaris demonstrate increased rates of MGD-related dry eye (Bayhan et al., 2016; Zakrzewska et al., 2023). Clinical observations further describe increased bacterial conjunctivitis and blepharitis incidence during retinoid therapy (Neudorfer et al., 2012). Direct effects of retinoids on the ocular microbiome remain uninvestigated, but these findings suggest an important research direction.

### 4.4 Essential oils

Tea tree oil (TTO) and its active compound terpinene-4-ol (T4O) are promoted in eyelid hygiene products for their antimicrobial properties against *Demodex* mite-associated blepharitis (Tighe et al., 2013; Wang et al., 2024).

However, T4O causes dose- and time-dependent decreases in immortalized HMGECs (Chen et al., 2020), and a case report documented bilateral corneal epithelial damage following use of 50% TTO solution (Tharmarajah and Coroneo, 2021). As corneal epithelial compromise may facilitate downstream microbial instability, the marketing of TTO products for eyelid and eyelash use represents a concern for MGD and associated dysbiosis.

### 4.5 Formulation context and real-world exposure relevance

Most available evidence linking cosmetic ingredients to ocular surface microbiome alterations is derived from *in vitro studies* using isolated compounds (**Table 1**). These conditions may not accurately reflect human use and exposure. Product formulations may demonstrate varying ingredient concentrations and human use likely has varying duration of contact between leave-on and rinse-off products. These variations may substantially influence microbial and epithelial responses. It is important to recognize that ocular effects have been observed at concentrations below regulatory reporting thresholds, underscoring the need for further labeling regulations (Chen et al., 2018). Future studies should incorporate standardized exposure models that account for real-world cosmetic exposure. This would give a more real-world assessment of the clinical significance of cosmetically associated dysbiosis as it relates to periocular cosmetics.

## 5 Links between dysbiosis and ocular surface disease

### 5.1 Meibomian gland dysfunction

MGD is associated with increased *Staphylococcus aureus* and decreased coagulase-negative *Staphylococcus*, *Corynebacterium*, and *Streptococci* (Watters et al., 2017). At the phylum level, MGD correlates with increased *Firmicutes* and *Proteobacteria* and decreased *Actinobacteria*. Severity of MGD is an important variable: significant α-diversity differences emerge between moderate and severe MGD groups, but not between mild MGD and healthy cohorts (Dong et al., 2019). IL-17A is significantly elevated in severe MGD, alongside pro-inflammatory cytokines IL-8, IL-6, IFN-γ, and ICAM-1 (Wu et al., 2020). **Figure 2** demonstrates how MGD leads to changes in ocular microbiome and increases the presence of proinflammatory cytokines and interleukins.

**Figure 2:**
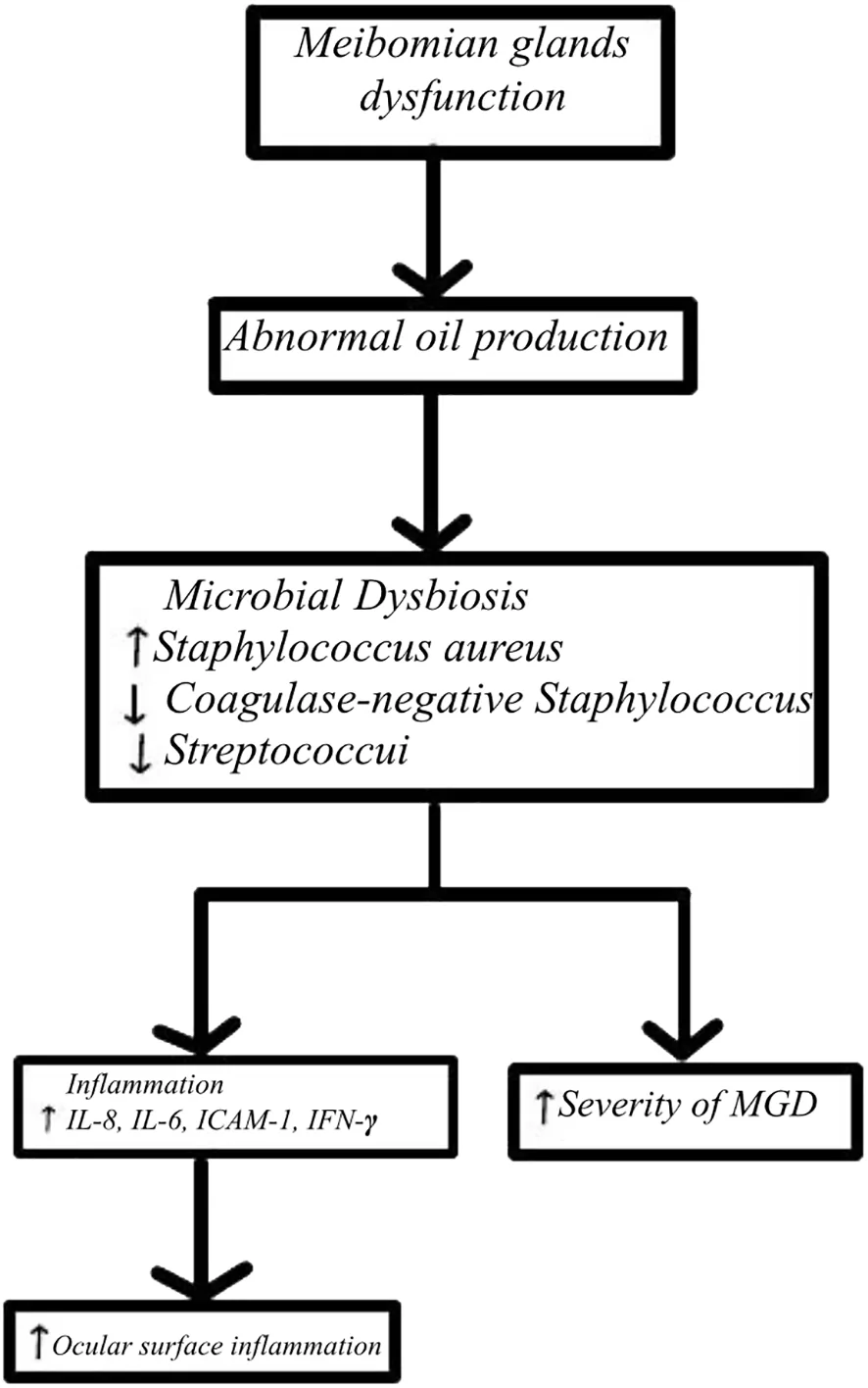
Associated ocular microbiome dysbiosis contributing to meibomian gland dysfunction (MGD). MGD is associated with microbiome community changes and increases in inflammatory markers: (1) meibomian gland dysfunction due to gland blockage or medication; (2) decreased quality and quantity of oil secretion; (3) disrupted ocular microbiome homeostasis (e.g., *S. aureus* increase, coagulase-negative *Staphylococcus* and *Streptococci* decrease); (4) increased pro-inflammatory cytokines (IL-8, IL-6, IFN-γ); (5) greater microbiome disruption correlating with MGD severity; and (6) increased ocular surface inflammation.

### 5.2 Blepharitis

Blepharitis has been associated with elevated *Cutibacterium acnes*, *Corynebacterium* spp., and *Staphylococcus aureus* (Benkaouha et al., 2015; Zhu et al., 2018). Patients with higher *Demodex folliculorum* counts show higher bacterial colony counts, particularly *C. acnes*, suggesting that mite overgrowth may amplify bacterial dysbiosis (Zhu et al., 2018). Importantly, blepharitis-associated microbiome shifts vary by geographic population, underscoring the role of environmental, dietary, and lifestyle factors (Wang et al., 2021). Across all populations studied, dysbiosis was consistently observed as blepharitis severity increased. An animal model using stat6vt mice — which spontaneously develop blepharitis — demonstrated that Th2 immune dysregulation (elevated IL-4, IL-5, IL-13) may represent a genetic driver (Turner et al., 2014). The microbiome changes observed in blepharitis are illustrated in **Figure 3**.

**Figure 3:**
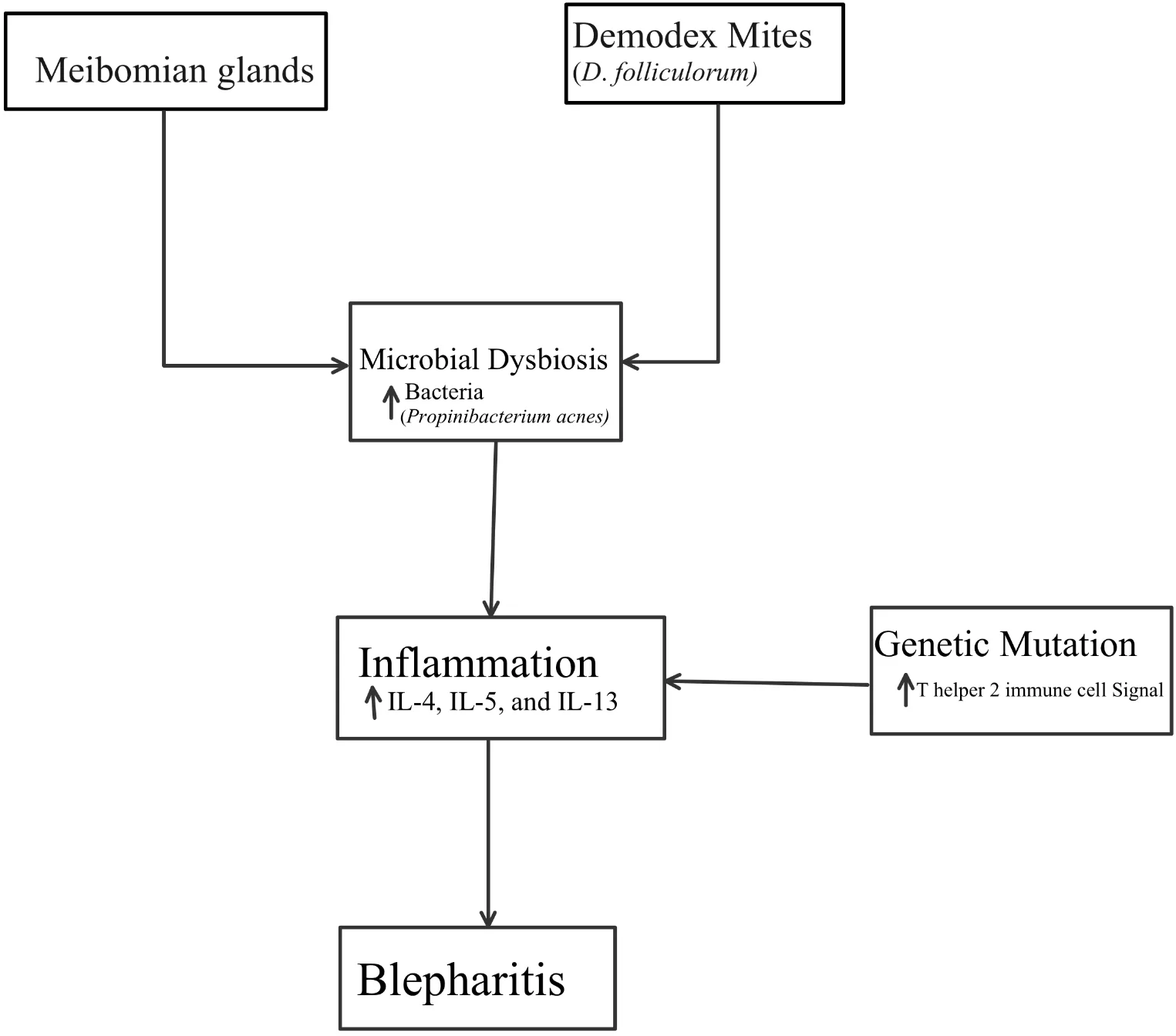
Demodex–dysbiosis–blepharitis axis. Proposed pathway linking *Demodex* mite overgrowth to blepharitis via ocular surface dysbiosis and inflammation. *D. folliculorum* overgrowth is associated with increased *Cutibacterium acnes (formerly Propionibacterium acnes)* and upregulation of inflammatory cytokines, amplified by possible genetic Th2 immune dysregulation.

### 5.3 Dry eye disease

Dry eye disease (DED) patients exhibit elevated *Corynebacterium tuberculostearicum* on the conjunctiva and increased *Propionibacteriaceae* on eyelid skin, compared to healthy controls (Schlegel et al., 2023). At the phylum level, DED and healthy patients share similar taxonomic compositions, with divergence evident at the genus level (Gupta et al., 2023). Differences in DED-associated microbiome shifts across studies conducted in Switzerland, India, and China suggest that climate and diet modulate ocular microbial composition, reinforcing the multifactorial nature of the disease. **Figure 4** summarizes the microbial shifts associated with DED.

**Figure 4:**
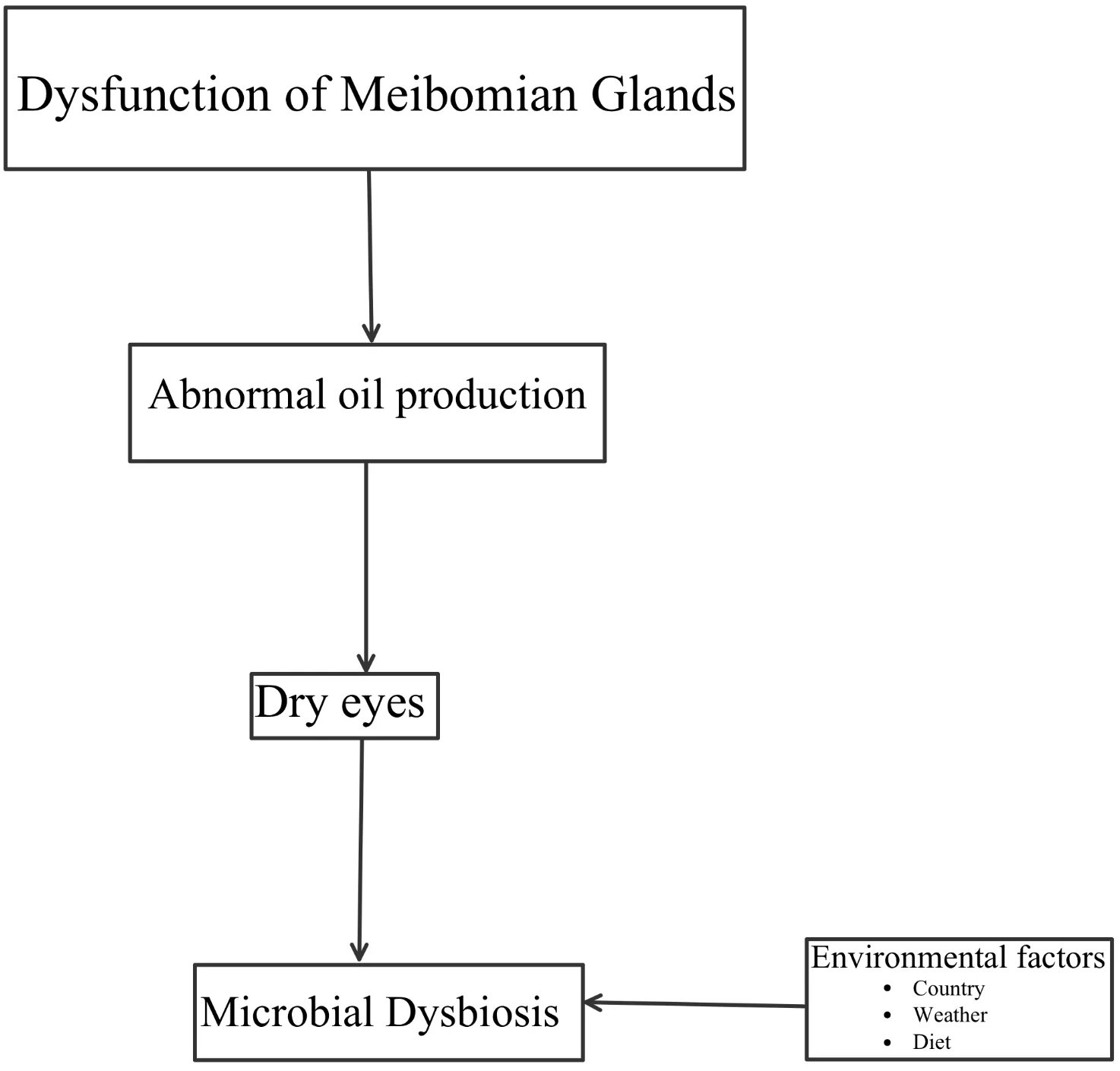
Dry eye-associated ocular surface dysbiosis. Meibomian gland dysfunction alters meibum quality/quantity and destabilizes the tear film, resulting in dry eye formation and increased *Corynebacterium tuberculostearicum* on the conjunctiva. Environmental factors further modulate microbial composition and disease severity.

## 6 Experimental models and research tools

### 6.1 Sampling techniques

Studying the ocular surface microbiome in humans requires representative, minimally contaminated specimens from the eyelid margin, conjunctiva, and tear film. Conjunctival swabs using nylon-flocked materials are commonly employed; swab material, pressure, and duration all influence sample quality (Chowdhary et al., 2023). Tear test strips are useful for microbiological studies related to DED, while conjunctival swabs are better suited for infectious disease studies (Chen et al., 2023). **Figure 5** summarizes the experimental framework used in ocular microbiome research, with selection of mode, exposure to the microbiome of a stimulating environment, evaluation of response, and measured impact on ocular health.

**Figure 5:**
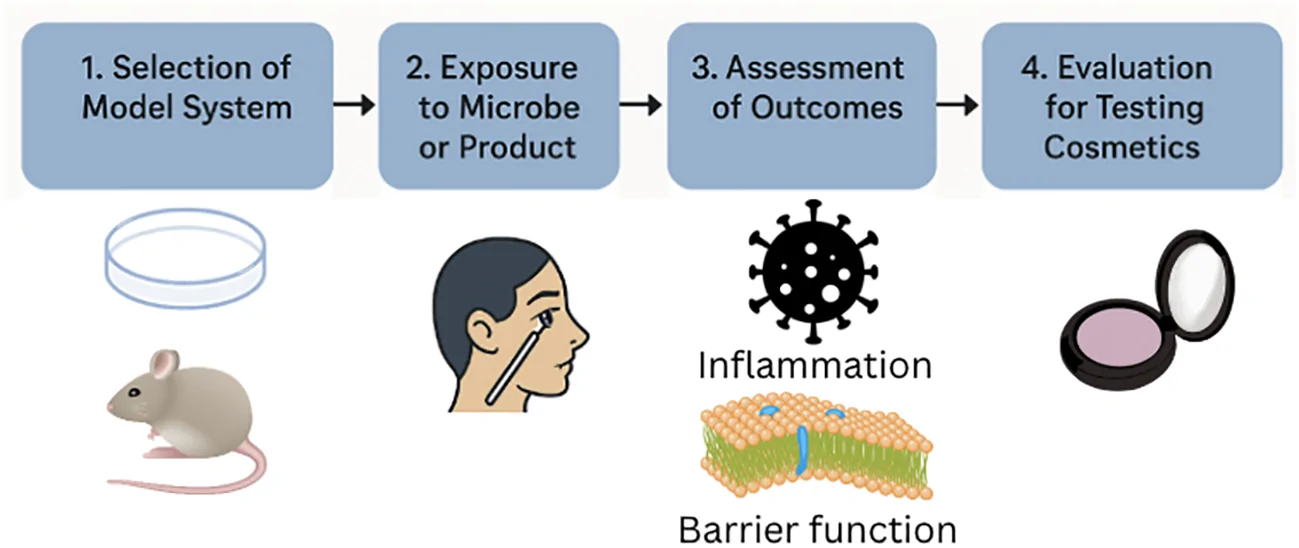
Ocular surface microbiome research framework. This diagram illustrates the experimental research procedure: (1) choosing the model system (e.g., *in vitro*, animal, or human sampling); (2) exposure to external agents or microbial stimuli (e.g., cosmetics, preservatives); (3) evaluating results of exposure (e.g., inflammation or barrier integrity); and (4) using results to assess how commercial products affect ocular health.

### 6.2 *In vitro* epithelial co-culture models

Three-dimensional co-culture systems combining rabbit conjunctival epithelial cells and lacrimal gland cell spheroids create both mucin-like and aqueous tear film layers that mirror the physical and biochemical barriers encountered by microbes at the ocular surface (Lu et al., 2017). IL-1β exposure in such co-cultures leads to epithelial thinning and goblet cell depletion, mimicking DED pathology (Lu et al., 2017). Limitations include reliance on non-human cell lines and absence of dynamic factors such as blinking or vascularization.

### 6.3 Animal models

Germ-free and antibiotic-treated mice demonstrate how microbial colonization influences mucosal homeostasis, including ocular surfaces (St Leger et al., 2017). IL-17-deficient and TLR-4-deficient (Toll-like receptor 4) knockout models are used to dissect molecular mechanisms of ocular immunity (Li et al., 2018). Limitations include anatomical differences (the murine nictitating membrane), reduced microbial diversity relative to humans, and significant ethical and technical challenges in low-volume sampling (Basu et al., 2024).

### 6.4 Sequencing technologies

16S rRNA gene sequencing is cost-effective and widely used for taxonomic profiling, with reliable genus-level resolution. However, its low species-level discriminating power and susceptibility to primer bias are significant limitations in low-biomass samples (Winand et al., 2019; Park et al., 2021). Shotgun metagenomics enables comprehensive functional profiling, including identification of antibiotic resistance genes (ARGs). In healthy individuals, 68.5% of ocular surface isolates carried ARGs conferring resistance to beta-lactams, macrolides, aminoglycosides, and tetracyclines (Dong et al., 2022). In keratitis patients with repeated antibiotic exposure, shotgun metagenomics identified enrichment of virulence factors and pathogenesis-related pathways (Kang et al., 2022).

Contamination control in low-biomass samples is critical and includes randomized sample processing, tracking of reagent batches, use of negative controls, and confirmation of unexpected findings via complementary methods such as culture or FISH (fluorescence *in situ* hybridization) (Salter et al., 2014). Metabolomics and metatranscriptomics represent emerging tools for capturing real-time microbial activity, though metatranscriptomics has not yet been applied directly to the human ocular surface microbiome. **Figure 6** summarizes the evolution of ocular surface techniques research tools, from traditional culture-based techniques to modern-day 16S rRNA genomic sequencing and shotgun metagenomics, while highlighting emerging tools that may shape future research directions.

**Figure 6:**
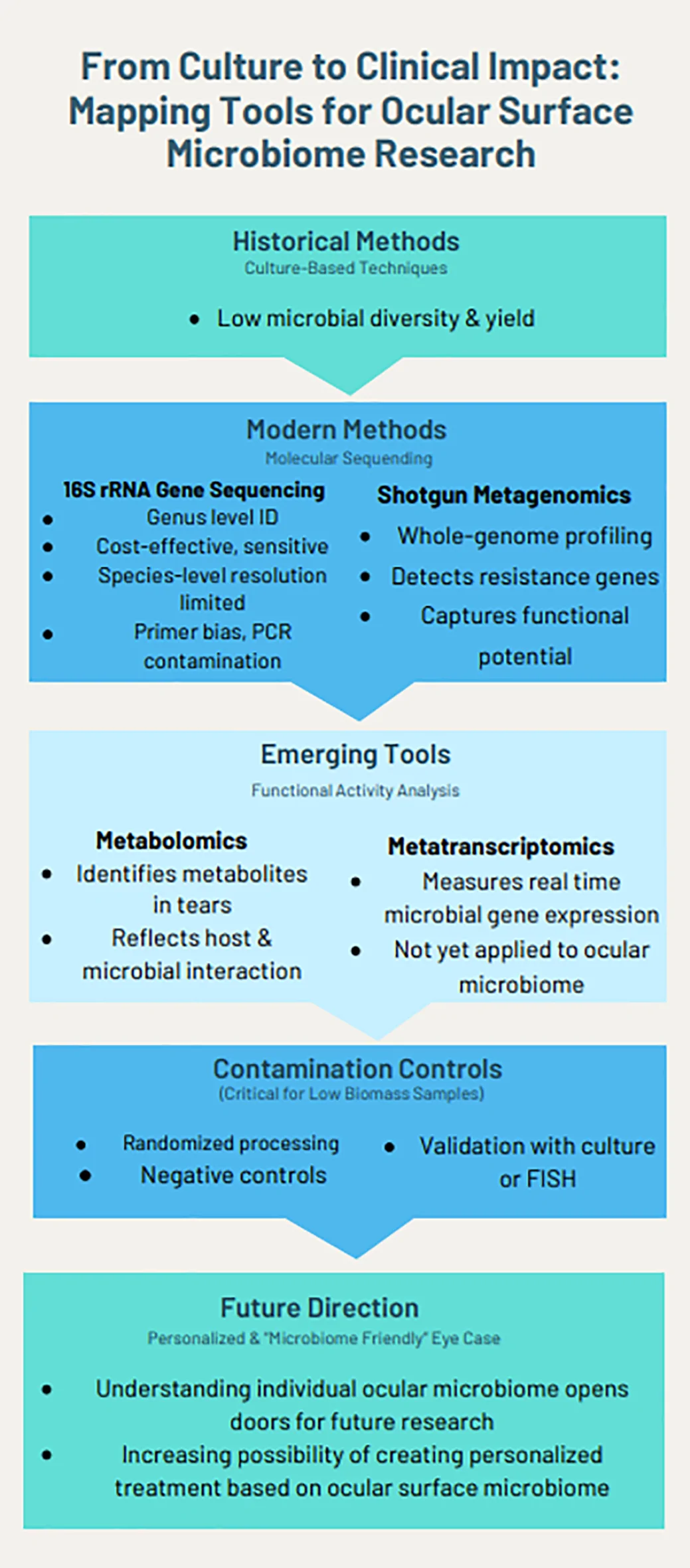
Overview of research tools used to characterize the ocular surface microbiome. Past culture-based techniques yielded few microorganisms. Modern biogenomic tools have characterized a broader microbiome community on the ocular surface. Future research is needed to determine variation of the ocular surface microbiome.

**Figure 7:**
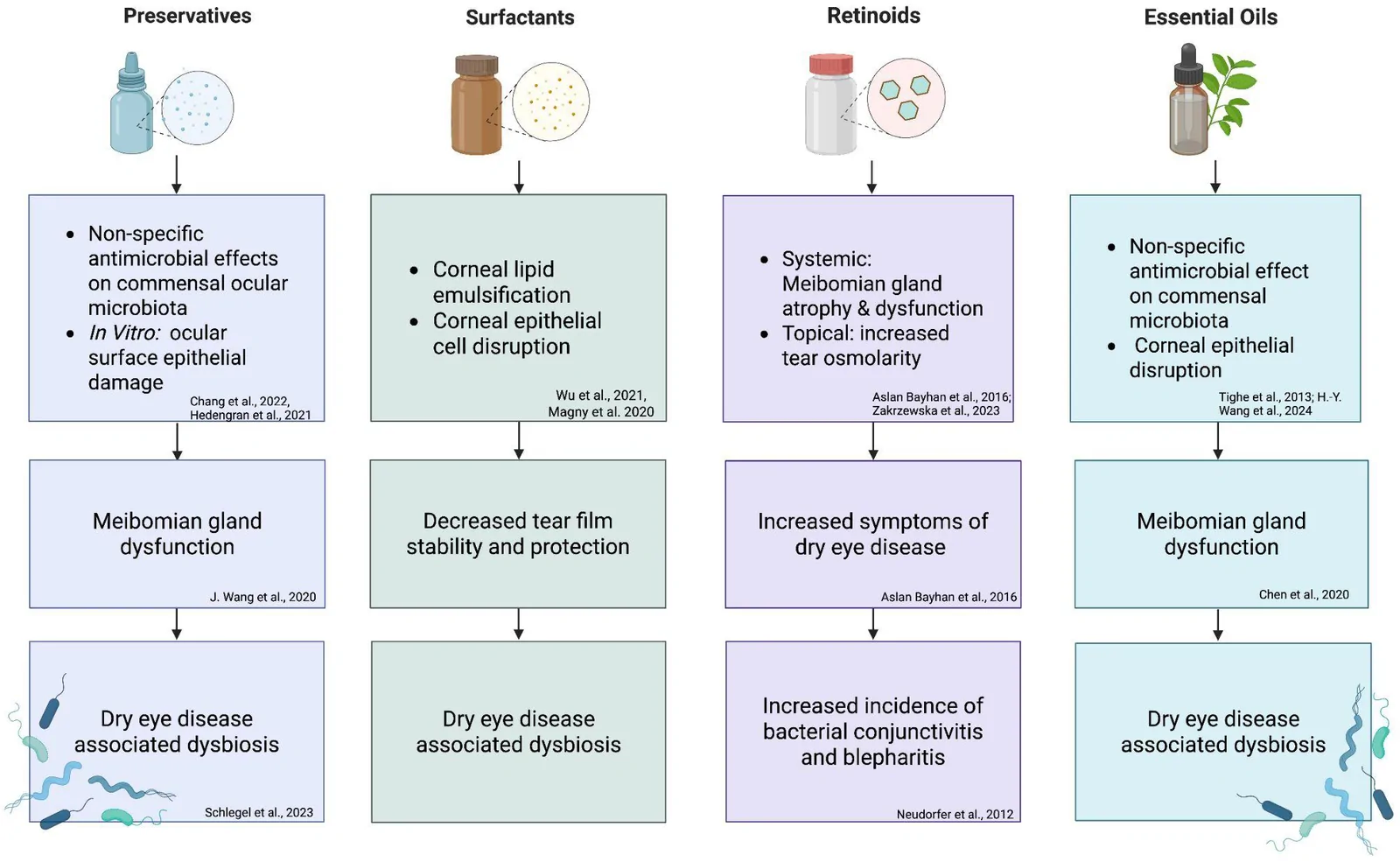
Effects of skincare and cosmetic ingredients on ocular surface dysbiosis. This schematic illustrates direct and indirect effects of cosmetic and skincare ingredients (preservatives, surfactants, retinoids, and essential oils) leading to dry eye disease and associated ocular surface dysbiosis.

**Figure 8:**
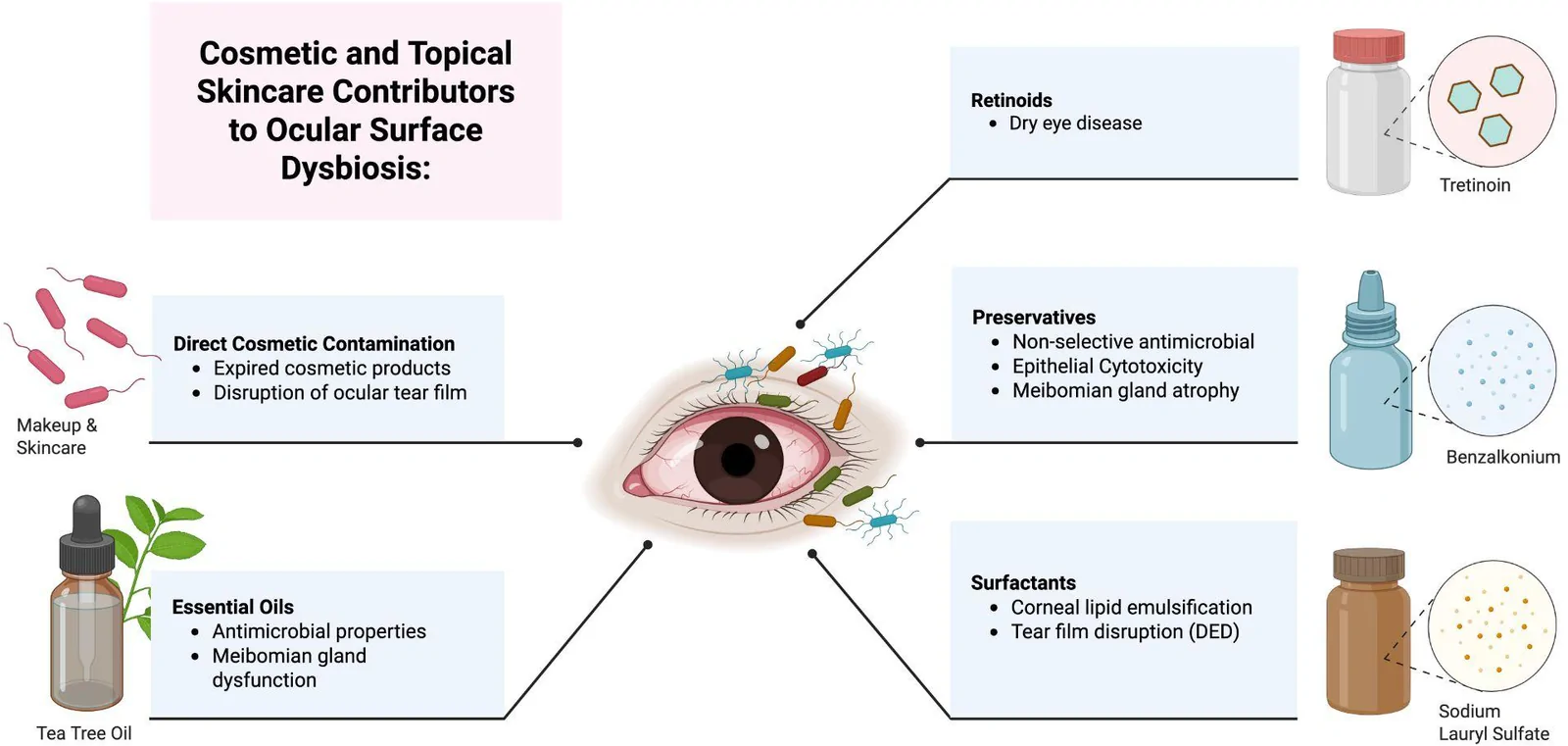
Cosmetic and topical skincare contributors to ocular surface dysbiosis. This diagram demonstrates the complex relationship of contributing factors leading to both direct and indirect disruptions of the ocular surface microbiome.

## 7 Regulatory landscape and research gaps

Under FDA guidelines (21 CFR 701.3(f)(2)), cosmetic manufacturers must list ingredients above 1% concentration in descending order, while those below 1% can be listed in any order. Specific concentrations are not required to be disclosed, even though ocular surface effects have been documented at sub-1% concentrations (Ko et al., 2018).

Unlike pharmaceuticals, cosmetics do not require pre-market approval (except some color additives), and no mandatory ocular-specific safety testing is required.

Some companies voluntarily pursue “Ophthalmologist-Tested” or “Eye Safe” labeling, but these standards are not standardized and do not account for downstream effects on the ocular microbiome. Under the Modernization of Cosmetics Regulation Act (MoCRA) of 2022, the FDA can now recall misbranded products, but cosmetic companies are not required to include ocular-specific warnings even for products likely to migrate to the ocular surface. Thus public-awareness strategies emphasizing the disposal of expired products, avoidance of product sharing, and improved ingredient literacy may help improve ocular health outcomes. Several online resources, including the Environmental Working Group’s Skin Deep database, provide consumers with accessible information about cosmetic ingredients and product safety profiles ( (Environmental Working Group, n.d)). While the large number and complexity of cosmetic ingredients make comprehensive consumer education challenging, increasing awareness of ingredients that may pose a higher risk could support more informed usage. Such efforts may ultimately promote healthier periocular and ocular surface practices.

In addition, cosmetic regulations in many countries address product safety, quality, risk assessment, and labeling requirements. However, substantial regulatory differences exist across regions. The European Union and Canada are generally regarded as having more stringent standards for ocular cosmetics than the United States (Sullivan et al., 2023). Efforts to promote greater international regulatory uniformity have emerged through the International Cooperation on Cosmetic Regulation (ICCR), an annual forum that includes cosmetic regulatory authorities from the United States, Canada, Japan, the European Commission, Brazil, Taiwan, and the Republic of Korea (Sullivan et al., 2023). Although the ICCR addresses a range of issues, including cosmetic allergens and safety assessment, its recommendations are voluntary and participation includes both regulatory authorities and industry representatives.

Key research gaps include: (1) absence of a consensus healthy ocular microbiome baseline; (2) over-reliance on*in vitro* models that do not replicate real-world ocular conditions; (3) a lack of longitudinal and dose–duration studies — the available literature does not establish how the duration or cumulative quantity of cosmetic use correlates with the magnitude of microbiome disruption, nor how frequently subclinical dysbiosis progresses to overt, symptomatic ocular surface disease (an important distinction, as sequencing-detected microbiome shifts may not always be clinically significant); and (4) insufficient population diversity, which limits generalizability across genetic and cultural cosmetic practices.

## 8 Conclusions

This scoping review synthesizes evidence demonstrating that periocular skincare and cosmetic products — through preservatives, surfactants, retinoids, and essential oils — disrupt the ocular surface microbiome via direct microbial depletion, epithelial cytotoxicity, and tear film lipid emulsification (**Figures 7**, **8**). These disruptions contribute to MGD, blepharitis, and evaporative dry eye, all of which exhibit characteristic dysbiotic profiles. It should be emphasized, however, that a substantial portion of this mechanistic evidence derives from *in vitro* and animal models, and the threshold at which subclinical microbiome shifts translate into clinically meaningful disease remains undefined. Regulatory and methodological gaps limit our current understanding. Future work should prioritize: longitudinal study designs that relate cumulative exposure duration to clinical outcomes; functional metagenomic approaches (16S rRNA, shotgun metagenomics, metabolomics, metatranscriptomics); ingredient-level *in vitro* testing pipelines that incorporate realistic formulation and exposure context; and diversified study populations. Clinicians evaluating patients with ocular surface disease should incorporate periocular product use history as a standard component of assessment, and broader public-awareness efforts around expired-product use and ingredient literacy may help reduce avoidable exposure.

## Data Availability

The original contributions presented in the study are included in the article/supplementary material. Further inquiries can be directed to the corresponding author.
